# Association of Diaphragm Involvement Assessed by Ultrasound With Disease Severity in Facioscapulohumeral Muscular Dystrophy

**DOI:** 10.1002/jcsm.70057

**Published:** 2025-09-12

**Authors:** Xiang Xu, Fuze Zheng, Xin Lin, Liangliang Qiu, Long Chen, Jing Xu, Li Kang, Jie Chen, Liulei Wu, Ying Zheng, Minghui Zeng, Xiaodan Lin, Qifang He, Li Chen, Feng Lin, Ning Wang, Minting Lin, Guorong Lyu, Zhiqiang Wang

**Affiliations:** ^1^ Department of Ultrasound, the First Affiliated Hospital Fujian Medical University Fuzhou China; ^2^ Department of Ultrasound, National Regional Medical Center, Binhai Campus of the First Affiliated Hospital Fujian Medical University Fuzhou China; ^3^ Department of Neurology and Institute of Neurology of the First Affiliated Hospital, Institute of Neuroscience, and Fujian Key Laboratory of Molecular Neurology Fujian Medical University Fuzhou China; ^4^ Department of Neurology, National Regional Medical Center, Binhai Campus of the First Affiliated Hospital Fujian Medical University Fuzhou China; ^5^ Department of Respiratory and Critical Care Medicine Pulmonary Function Test Center, Respiratory Disease Research Institute, the First Affiliated Hospital Fujian Medical University Fuzhou China; ^6^ Department of Respiratory and Critical Care Medicine Pulmonary Function Test Center, National Regional Medical Center, Binhai Campus of the First Affiliated Hospital Fujian Medical University Fuzhou China; ^7^ Department of Ultrasound, the Second Affiliated Hospital Fujian Medical University Quanzhou China; ^8^ Quanzhou Medical College Quanzhou China

**Keywords:** diaphragm involvement, disease severity, facioscapulohumeral muscular dystrophy type 1 (FSHD1), respiratory involvement, ultrasound

## Abstract

**Background:**

Respiratory involvement is a comorbidity that should not be overlooked in clinical practice in facioscapulohumeral muscular dystrophy type 1 (FSHD1), with a reported association for severe disease outcomes such as wheelchair dependency. However, patients with FSHD1 can have inaccurate ventilatory function assessment results, owing to facial muscle involvement. Additionally, the association between diaphragm involvement and disease severity in FSHD1 patients remains uncertain. This study aims to assess diaphragm involvement using ultrasound technology and to assess potential associations of diaphragm involvement with respiratory involvement and disease severity.

**Methods:**

This prospective, observational, case–control study enrolled genetically confirmed FSHD1 patients from the Chinese FSHD1 cohort and control participants (matched with FSHD1 patients at a ratio of 2:1 based on gender, age at examination and height) between January 2021 and February 2025. Ultrasound examination of the diaphragm and pulmonary function tests were performed to evaluate respiratory involvement in both FSHD1 patients and paired controls.

**Results:**

The final analytical sample included 109 patients: 81 patients (median [IQR] age, 33 [23–43] years; 33 [40.7%] female) and 162 control participants (median [IQR] age, 31 [23–45] years; 66 [40.7%] woman) in the exploration cohort, and 28 patients in the validation cohort. Ultrasound parameters of the right hemidiaphragm for diaphragm excursion velocity (*V*_VS_), maximal relaxation rate of the diaphragm (ECHO‐MRR) and thickness of the diaphragm at total lung capacity (*Th*_TLC_) displayed significant differences between the FSHD1 groups with vs. without restrictive ventilatory defect (RVD). A multivariate model (including variables of sex, age at examination, D4Z4 RUs, *V*_VS_ and ECHO‐MRR) efficiently identified RVD in the ROC curve analysis with an AUC of 0.943 (0.896–0.989). In the validation cohort, applying the cut‐off values for *V*_VS_ derived from ROC analysis to identify RVD in FSHD1 patients, results showed a high true positive rate of 80.0% and a true negative rate of 94.4%. Multivariate Cox regression analyses indicated low *V*_VS_ and low ECHO‐MRR were independently associated with early lower extremity involvement in FSHD1, with adjusted hazard ratios (aHRs) (95% CI) of 2.353 (1.356–4.085) and 2.039 (1.186–3.504), respectively. Multivariate linear regression models indicated that lower *V*_VS_ (*β* = −1.686) and ECHO‐MRR (*β* = −1.761) of the right diaphragm were significantly associated with higher age‐corrected CSS in FSHD1 patients.

**Conclusions:**

Ultrasound parameters, including *V*_VS_ and ECHO‐MRR, are informative for assessing diaphragm involvement and respiratory involvement in FSHD1. This knowledge establishes diaphragm ultrasound parameters as potential biomarkers of FSHD1 disease severity in upcoming clinical trials.

## 1 Introduction

Facioscapulohumeral muscular dystrophy type 1 (FSHD1) is one of the most common forms of muscular dystrophy, characterized by progressive weakness in the face and upper extremities, gradually leading to weakness in the respiratory muscles, abdominal muscles, and lower extremities [1, 2]. In FSHD1, respiratory involvement is increasingly recognized as recent studies have reported, though it remains subclinical during the early stage of the disease [2, 3]. Moreover, respiratory involvement has been associated with severe outcomes such as wheelchair dependency [4]. Genetically, FSHD1 is caused by contraction of macrosatellite D4Z4 repeat units (RUs) located on chromosome 4 [5]. Notably, lower D4Z4 RUs have been associated with increased disease severity and with severe respiratory involvement [6, 7].

The diaphragm is the main inspiratory muscle, undertaking more than 90% of the ventilation during inspiration [8], and diaphragm involvement has been identified as a main risk factor for restrictive ventilatory defect (RVD) in FSHD1 [4, 5]. For respiratory involvement assessment in FSHD1 patients, the commonly used method of pulmonary function tests (PFTs) can yield inaccurate values (specifically for the FVC and FEV1 manoeuvres); this is because of facial muscle weaknesses in FSHD1 patients (presenting in ~74% of FSHD1 cases [9]). Patients are often unable to seal the lips around the mouthpiece, leading to leakage at the mouth [9]. Consequently, conventional PFTs cannot reliably assess respiratory function in this population unless technical adjuncts (e.g., manual mouth support or vacuum‐seal masks) are employed [10, 11, 12].

There are alternatives to PFT for respiratory involvement assessment [13, 14, 15]. For example, studies of healthy subjects have used ultrasound to assess diaphragm excursion and thickness and have reported that the obtained values correlate closely with PFT parameters [16]. Moreover, the utility of diaphragmatic ultrasound in critically ill patients for assessing the withdrawal of mechanical ventilation is now widely established, and diaphragm involvement detection based on ultrasound data is now viewed as a reliable predictor for impaired pulmonary function in multiple neuromuscular disorders (NMD) [17, 18]. However, there are limited data on the potential association between ultrasound‐based diaphragm assessment and respiratory function in FSHD1 [19]. Additionally, whether diaphragm involvement is associated with disease severity remains unknown.

Here, we performed a prospective and observational case–control study based on the Chinese FSHD1 cohort to investigate the potential association of diaphragm involvement assessed by ultrasound with respiratory involvement of FSHD1. Furthermore, we assessed the potential association of diaphragm involvement with disease severity and progression to lower extremities, offering clinicians a potential imaging biomarker for future clinical trials.

## 2 Methods

### 2.1 Study Design and Participant Recruitment

This prospective and observational case–control study was conducted at the First Affiliated Hospital of Fujian Medical University in Fuzhou, China, between January 2021 and February 2025. Eligible participants included FSHD1 patients, recruited from the Chinese FSHD1 cohort (Clinical Trial Identifier: NCT04369209) [20], and control participants from the First Affiliated Hospital of Fujian Medical University. The study flowchart is shown in Figure S1. For the exploration cohort, patients with FSHD1 were diagnosed according to genetic diagnostic criteria of D4Z4 RUs as described in previous studies [7, 21, 22]. Participants in the control group, who were individually matched with FSHD1 patients at a ratio of 2:1 based on gender, age at examination (±3 years) and height (±2 cm), were recruited from among genetically confirmed healthy family members of patients and healthy controls from a health centre in our hospital. Exclusion criteria were applied as follows: participants who had an acute illness during a month before their visit, those with other neuromuscular diseases, sarcopenia, ischemic/haemorrhagic stroke, abnormal cardiac function, respiratory diseases and a history of thoracic or abdominal surgery. No patients were receiving ventilator therapy at the time of enrolment. PFTs were performed for both FSHD1 patients and controls and were used to assess RVD status. The final exploration cohort consisted of three groups: (1) FSHD1 with RVD, (2) FSHD1 without RVD and (3) control participants. A validation cohort of 28 FSHD1 patients was established to assess the sensitivity of diaphragm ultrasound parameters for identifying RVD in FSHD1 patients.

This study was approved by the ethics committee for Medical Research of the First Affiliated Hospital of Fujian Medical University. Informed written consent was obtained from each patient and their parents or guardians before participation in the study.

### 2.2 Diaphragm Ultrasound Imaging Acquisition and Analysis

**FIGURE 1:**
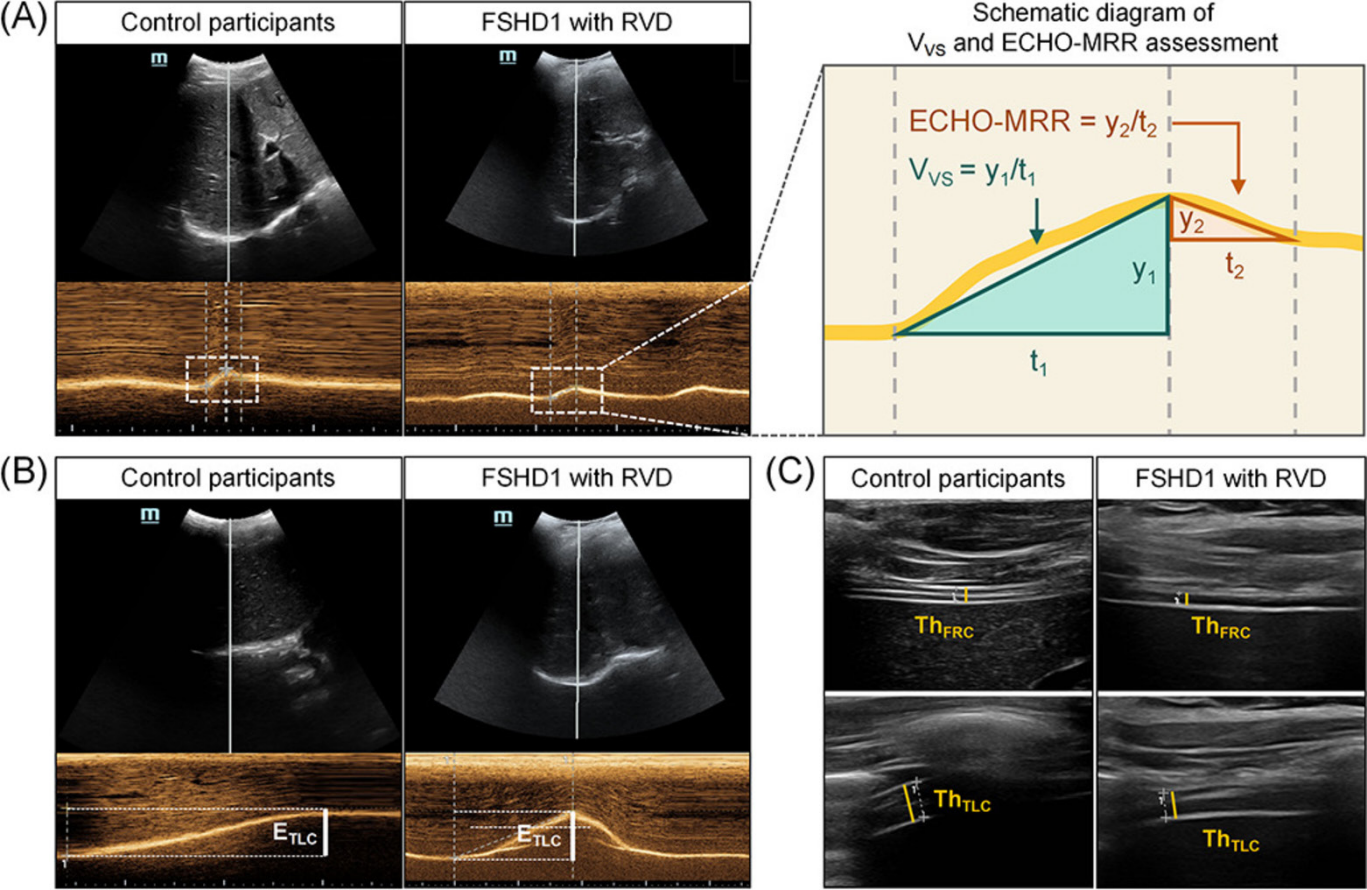
Schematic diagram of diaphragm ultrasound parameter measurements in control participants and FSHD1 patients with RVD. (A) *V*_VS_ was defined as the slope of the curve between the lowest and highest points (green continuous line) during maximum voluntary sniff on M‐mode ultrasound (*V*_VS_ = *y*_1_/*t*_1_, where *y*_1_ is the vertical dimension between the lowest and highest points and *t*_1_ is the time it takes to get from the lowest point to the highest point). ECHO‐MRR was defined as the slope of the initial steepest descending segment (red continuous line) during expiration phase of maximum voluntary sniff on the M‐mode ultrasound (ECHO‐MRR = *y*_2_/*t*_2_, where *y*_2_ was vertical dimension between the highest and the first inflection points and *t*_2_ was the time it takes to get from the highest point to the first inflection point). (B) *E*_TLC_ was defined as the vertical distance between the lowest and highest points of diaphragm displacement (white continuous line) at total lung capacity on M‐mode ultrasound. (C) *Th*_FRC_ is measured as the vertical distance of the diaphragm between the inner edge of the pleural line and the inner edge of the peritoneal line (yellow continuous line) at functional residual capacity on B‐mode. *Th*_TLC_ was measured as the vertical distance of the diaphragm between the inner edge of the pleural line and the inner edge of the peritoneal line (yellow continuous line) at total lung capacity on B‐mode.

All FSHD1 patients and control participants underwent diaphragm assessments performed by the examiners using identical equipment and methodologies. Diaphragm ultrasound imaging was performed using a scanning protocol as published previously [10, 23, 24]. Briefly, we assessed diaphragm function using M‐mode and B‐mode ultrasound imaging (Figure 1). Diaphragm ultrasound parameters included diaphragm excursion velocity during maximum voluntary sniffing (*V*_VS_), the maximal relaxation rate of diaphragm obtained with ultrasound (ECHO‐MRR), excursion amplitude of the diaphragm at total lung capacity (*E*_TLC_), thickness of the diaphragm at total lung capacity (TLC) (*Th*_TLC_), functional residual capacity (FRC) (*Th*_FRC_) and diaphragm thickening ratio (DTR) [10, 23, 24]. The DTR was calculated according to the following formula [23]:

$$\mathrm{DTR}={\mathrm{Th}}_{\mathrm{TLC}}/{\mathrm{Th}}_{\mathrm{FRC}}$$

According to two distinct functions of respiratory muscles: shortening (lung volume changes) and force development (pressure changes), we divided the ultrasonographic assessment parameters of the diaphragm into two categories representing the contractile and relaxant functions of the diaphragm: (1) shortening category parameters, excursion (*E*_TLC_) and thickness (*Th*_FRC_, *Th*_TLC_ and DTR), denoting lung volume changes, and (2) force category parameters, *V*_VS_ and ECHO‐MRR.

### 2.3 Clinical Assessments

All patients with FSHD1 were examined by the same neurologist (Z.Q.W.). Clinical data collected included the number of contractions of the macrosatellite D4Z4 RUs, muscle strength, disease severity, and age at onset (mainly obtained from patients' records or recollections) [20]. FSHD1 patients were stratified into three groups based on age at onset (AAO), according to a previous study [22]: (1) early onset group (AAO < 10 years), (2) typical onset group (10 ≤ AAO < 30 years) and (3) late onset group (AAO ≥ 30 years).

Muscle strength was assessed through Medical Research Council (MRC) score (the following muscle pairs: shoulder abductors, arm exorotation, shoulder adductors, elbow flexors, elbow extensors, wrist extensors, wrist flexors, gluteus, hip flexors, hip abductors, hip adductors, knee flexors, knee extensors, foot dorsal flexors and foot plantar flexors) [25]. Clinical severity was assessed by implementing specific scales: (1) FSHD clinical score (CS) [26], (2) FSHD clinical severity scale (CSS) and (3) age‐corrected CSS (ACSS), adjusted for the patient's age at examination according to the following formula [27]:

$$\left(\left[\mathrm{CSS}\times 2\right]/\mathrm{age}\;\mathrm{at}\;\text{examination}\right)\times 1000$$

### 2.4 PFTs

PFTs were performed in the seated position in all FSHD1 patients using an electronic spirometer (MasterScreen, Vyaire, Würzburg, Germany) [11, 12]. Auxiliary measures, including manual support and the use of face masks, were employed to ensure reliable PFT data obtained from FSHD1 patients with notable facial muscle weakness (particularly orbicularis oris involvement, *n* = 23). Additionally, PFTs were performed in the supine position for a subset of seven patients in the validation cohort; these data were used to examine potential differences between PFTs performed in the seated and supine positions. Forced vital capacity (FVC) and forced expiratory volume in the first second (FEV_1_) were measured in litres. At least five consecutive tests were performed until the best result was achieved and showed less than 10% variation from the preceding test. FVC was expressed as a percentage of the predicted value based on gender, height and age. FSHD1 patients with RVD were defined as patients with FVC < 80% predicted and a FEV1/FVC ratio of ≥ 70% predicted [14]. Mild RVD was defined as FVC 70%–80% predicted, moderate RVD as FVC 60%–70% predicted and moderate‐severe RVD as FVC 50%–60% predicted [11, 12]. FSHD1 patients were stratified into two subgroups, with and without RVD, based on PFTs [10].

### 2.5 Statistical Analysis

Continuous variables were summarized by mean (SD) or median (IQR), as appropriate. Categorical variables were presented by frequency and percentage (%). Comparisons of nonparametric continuous variables were performed using Mann–Whitney *U* tests (for two groups) or Kruskal–Wallis *H* tests (for > 2 groups; statistical significance value was adjusted by Bonferroni correction); categorical variables were compared using *χ* ^2^ tests (Fisher's exact test when the expected value was < 5). Bilateral diaphragm ultrasound parameters were compared using Wilcoxon signed‐rank tests. Correlations between variables were assessed using the Spearman correlation coefficient. Multivariate linear regression was performed to assess relationships between diaphragm ultrasound parameters and clinical outcome measures of disease severity.

Area under the receiver operating characteristic (ROC) curve (AUC) was used to assess the ability of diaphragm ultrasound parameters to identify RVD among FSHD1 patients. The corresponding cut‐off values of diaphragm ultrasound parameters were obtained from ROC analysis. Then, FSHD1 patients were stratified into groups according to cut‐off values: (1) FSHD1 patients with low *V*_VS_/ECHO‐MRR subgroup and (2) FSHD1 patients with high *V*_VS_/ECHO‐MRR subgroup.

Kaplan–Meier curves were used to compare the cumulative probability risk of lower extremity (LE) involvement between FSHD1 patients in the low and high *V*_VS_/ECHO‐MRR subgroups. A log‐rank test was used to determine any statistical differences in the Kaplan–Meier curves between the groups. Cox proportional hazards regression models were used initially to obtain adjusted hazard ratios (aHRs) and 95% confidence intervals (CIs) in the subgroup with RVD compared to the group without RVD at the outcome of LE involvement.

Statistical analyses were performed with SPSS 25.0 and GraphPad Prism 9.0. A *p* value of < 0.05 was considered statistically significant.

## 3 Results

### 3.1 Baseline Characteristics

A total of 271 participants were included in the current study, comprising 81 genetically confirmed FSHD1 patients and 162 control participants in the exploration cohort and 28 genetically confirmed FSHD1 patients in the validation cohort. Among the 81 patients with FSHD1 in the exploration cohort, 33 (40.7%) were female, with the median age at examination of 33 (23, 43) years and the median BMI of 20.8 (18.9, 22.6) kg·m^−2^. Genetically, the median number of the contracted D4Z4 RUs of FSHD1 patients was five RUs (range 2–10 RUs). In clinical assessments, the median age at onset was 15 years (range 1–50 years), the median FSHD CS score was 7.0 (range 1.0–15.0), and the median ACSS score was 175.5 (range 23.8–1000.0) (Table 1). In the control group matched with FSHD1 patients, 33 (40.7%) were female, with the median age at examination of 31 (23, 45) years. Both the median BMI of 22.6 (21.8, 23.1) kg·m^−2^ and weight of 62.0 (57.0, 67.0) kg in control participants were significantly higher than that in the FSHD1 patients (*p* < 0.0001).

**TABLE 1:** Baseline Characteristics of FSHD1 patients and control participants in the exploration cohort.

	FSHD1 patients			Control participants (*N* = 162)	*p* ^a^
	Total (*N* = 81)	FSHD1 with RVD (*N* = 41)	FSHD1 without RVD (*N* = 40)		
Demographics					
Male, *n* (%)	48 (59.3)	26 (63.4)	22 (55.0)	96 (59.3)	0.594
Age at examination, *y*, median (IQR)	33 (23, 43)	30 (18, 46)	34 (29, 43)	31 (23, 45)	0.254
Height, cm, median (IQR)	166 (159, 172)	164 (157, 173)	169 (161, 172)	166 (160, 171)	0.259
Weight, kg, median (IQR)	60.0 (51.0, 66.0)	56.0 (42.5, 65.0)	61.0 (55.3, 57.5)	62.0 (57.0, 67.0)	**0.014**
BMI, kg·m^−2^, median (IQR)	20.8 (18.9, 22.6)	20.2 (17.9, 22.6)	21.6 (20.4, 22.9)*	22.6 (21.8, 23.1)	**< 0.0001**
Bilateral diaphragm ultrasound parameters, median (IQR)					
Right hemidiaphragm					
*V*_VS_, mm/s	63.3 (50.9, 73.8)	52.0 (44.0, 62.6)###	73.2 (65.6, 90.0)***	89.0 (84.0, 93.3)	**< 0.0001**
ECHO‐MRR, mm/s	38.3 (24.1, 46.9)	24.8 (16.2, 37.3)###	44.9 (40.0, 49.4)***	65.0 (57.0, 71.0)	**< 0.0001**
*E*_TLC_, mm,	66.1 (56.1, 76.7)	61.7 (41.3, 70.4)##	67.8 (62.1, 80.1)	75.2 (70.0, 79.9)	**< 0.0001**
*Th*_FRC_, mm	1.1 (0.9, 1.3)	1.1 (0.9, 1.3)	1.1 (0.9, 1.3)***	1.4 (1.3, 1.6)	**< 0.0001**
*Th*_TLC_, mm	2.4 (2.0, 3.4)	2.1 (1.8, 2.5)##	3.0 (2.4, 3.5)***	3.7 (3.3, 4.3)	**< 0.0001**
DTR	2.4 (1.8, 2.8)	1.9 (1.5, 2.6)##	2.6 (2.3, 3.3)	2.6 (2.5, 2.8)	**< 0.0001**
Left hemidiaphragm					
*V*_VS_, mm/s	61.8 (43.7, 79.9)	44.5 (32.3, 59.6)###	72.6 (64.3, 87.0)**	86.5 (81.0, 91.0)	**< 0.0001**
ECHO‐MRR, mm/s	22.1 (14.0, 40.0)	17.2 (10.0, 23.3)#	39.5 (21.7, 43.9)***	63.0 (57.0, 69.0)	**< 0.0001**
*E*_TLC_, mm	57.6 (43.3, 70.8)	49.7 (37.0, 64.8)#	64.9 (53.2, 75.0)***	76.4 (70.7, 81.7)	**< 0.0001**
*Th*_FRC_, mm	1.0 (0.9, 1.2)	1.0 (0.8, 1.2)	1.1 (0.9, 1.2)***	1.3 (1.2, 1.5)	**< 0.0001**
*Th*_TLC_, mm	2.3 (1.9, 3.0)	2.0 (1.5, 2.7)##	2.8 (2.1, 3.5)***	3.7 (3.3, 4.0)	**< 0.0001**
DTR	2.3 (1.7, 2.9)	2.0 (1.5, 2.5)###	2.6 (2.1, 3.3)	2.7 (2.6, 2.8)	**< 0.0001**
Pulmonary function tests (PFTs), median (IQR)					
FVC%predicted	84.0 (70.5, 89.9)	70.5 (67.7, 72.8)###	90.0 (86.4, 99.6)	90.0 (87.0, 94.0)	**< 0.0001**
FEV1%predicted	81.0 (71.5, 87.0)	71.5 (68.0,78.6)###	86.5 (82.6, 93.4)	87.0 (83.0, 90.0)	**< 0.0001**
Restrictive ventilatory defect (RVD) grading, *n* (%)					
Mild RVD	25 (30.9)	25 (61.0)	0 (0.0)		
Moderate RVD	13 (16.0)	13 (31.7)	0 (0.0)		
Moderate‐severe RVD	3 (3.7)	3 (7.3)	0 (0.0)		
Ventilator therapy	0 (0.0)	0 (0.0)	0 (0.0)		
Genetic characteristics					
Number of contracted D4Z4 repeat units, median (IQR)	5 (4, 6)	5 (4, 6)	5 (4, 7)		
Clinical assessments					
Age at onset, *y*, median (IQR)	15 (10, 20)	13 (7, 20)	15 (11, 20)		
Early‐onset, *n* (%)	19 (23.5)	14 (34.1)#	5 (12.5)		
Medical Research Council (MRC) score, median (IQR)					
Upper extremity strength	56.0 (47.0, 61.0)	53.5 (43.5, 59.3)#	58.3 (49.6, 65.8)		
Lower Extremity strength	58.5 (47.0, 66.0)	49.0 (38.3, 60.3)###	65.5 (55.8, 69.9)		
Clinical severity/progression assessments, median (IQR)					
FSHD clinical score (CS, 0–15)	7.0 (5.0, 9.0)	8.0 (6.0, 10.0)##	6.0 (4.0, 8.0)		
Clinical severity scale (CSS, 0–5)	3.0 (2.5, 3.5)	3.5 (3.0, 4.0)###	2.5 (2.0, 3.0)		
Age‐corrected CSS (ACSS, 0–10 000)	176.5 (125.1, 283.5)	241.4 (150.1, 444.4)###	138.2 (111.6, 207.6)		
Wheelchair dependency, *n* (%)	2 (2.5)	2 (4.9)	0 (0.0)		

*Note:* Mann–Whitney *U* test (for two groups; values were adjusted by Bonferroni correction) or Kruskal–Wallis *H* test (for three groups); categorical variables were compared using *χ* ^2^ tests.

^a^ Comparisons among FSHD1 patients with RVD, FSHD1 patients without RVD, and controls. Values in bold indicate *p* values of < 0.05 (Kruskal–Wallis *H* test).

^b^ Comparison of FSHD1 without RVD group and control group (Mann–Whitney *U* test); *p < 0.05; **p < 0.01; ***p < 0.001.

^c^ Comparison of FSHD1 with RVD group and FSHD1 without RVD group (Mann–Whitney *U* test); #*p* < 0.05; ##*p* < 0.01; ###*p* < 0.001.

### 3.2 Distinct Hemidiaphragm Weakness in FSHD1 Compared to Controls

Generally, we found that all diaphragm ultrasound parameter values of the bilateral diaphragm were significantly lower in FSHD1 patients compared to control participants, revealing the diaphragm involvement in FSHD1 (all *p* < 0.0001) (Table S1). In addition, the PFT parameters of FVC% predicted and FEV1 % predicted were significantly lower in FSHD1 patients compared to control participants (both *p* < 0.0001).

We next investigated the hemidiaphragm weakness characteristics in FSHD1 patients compared to controls (Table 2). Among the control participants, the *V*_VS_, ECHO‐MRR and *Th*_TLC_ values were significantly higher in the right hemidiaphragm compared to the left (all *p* < 0.05), whereas the *E*_TLC_ value was significantly lower in the right hemidiaphragm (all *p* < 0.05). Among FSHD1 patients, no significant differences were observed in *V*_VS_ (*p* = 0.183) or *Th*_TLC_ (*p* = 0.084) between the bilateral diaphragms. Moreover, compared to control participants, we observed a contrasting trend where the *E*_TLC_ value in the right hemidiaphragm was significantly higher than that in the left hemidiaphragm among FSHD1 patients (all *p* < 0.05).

**TABLE 2:** Comparison of bilateral diaphragm ultrasound parameters of FSHD1 and control participants.

Parameters	FSHD1 patients (*N* = 81)			Control participants (*N* = 162)		
	Right	Left	*p* ^a^	Right	Left	*p* ^a^
*V*_VS_, mm/s, mean (SD)	64.3 (18.1)	61.6 (23.0)	0.183	89.0 (7.1)	85.4 (6.9)	**< 0.0001**
ECHO‐MRR, mm/s, mean (SD)	35.7 (15.9)	28.1 (16.4)	**< 0.0001**	63.6 (10.3)	62.4 (8.4)	**0.040**
*E*_TLC_, mm, mean (SD)	65.4 (20.4)	57.1 (21.4)	**0.003**	75.9 (8.3)	76.5 (8.7)	**0.009**
*Th*_FRC_, mm, mean (SD)	1.1 (0.3)	1.1 (0.3)	**0.005**	1.4 (0.3)	1.4 (0.2)	**< 0.0001**
*Th*_TLC_, mm, mean (SD)	2.6 (0.9)	2.5 (1.0)	0.084	3.9 (0.8)	3.7 (0.6)	**< 0.0001**
DTR	2.4 (0.7)	2.4 (0.9)	0.951	2.7 (0.3)	2.7 (0.2)	0.459

*Note:* Values in bold indicate *p* < 0.05.

^a^ Two related samples Wilcoxon signed rank test was used.

### 3.3 Respiratory Involvement Assessed by Diaphragm Ultrasound Parameters in FSHD1

Among the exploration cohort, 41 patients had an RVD, including 25 (61.0%) with mild RVD, 13 (31.7%) with moderate RVD and 3 (7.3%) with moderate‐severe RVD. Diaphragm ultrasound parameters, including *V*_VS_, ECHO‐MRR, and *Th*_TLC_, were significantly lower in FSHD1 patients with RVD than in those without RVD (all *p* < 0.05) (Table 1). Furthermore, FSHD1 patients without RVD exhibited significantly lower bilateral diaphragm ultrasound parameters, such as *V*_VS_, ECHO‐MRR, *Th*_FRC_ and *Th*_TLC_, compared to control participants (all *p* < 0.001), whereas no significant differences were observed in PFT parameters of FVC% predicted and FEV1 % predicted (both *p* > 0.05) between these two subgroups (Table 1).

We then investigated correlations between bilateral diaphragm ultrasound parameters and PFT parameters (Table S2). The ultrasound parameters of *V*_VS_ (Spearman's *r* = 0.639, *p* < 0.0001), ECHO‐MRR (Spearman's *r* = 0.521, *p* < 0.0001), and *Th*_TLC_ (Spearman's *r* = 0.502, *p* < 0.0001) in the right hemidiaphragm were moderately positively correlated to FVC% predicted. The *E*_TLC_ (Spearman's *r* = 0.335, *p* < 0.0001) and DTR (Spearman's *r* = 0.413, *p* < 0.0001) in the right hemidiaphragm showed mild positive correlations with FVC% predicted. Mild positive correlations were also found between the left hemidiaphragm ultrasound parameters and FVC% predicted.

**FIGURE 2:**
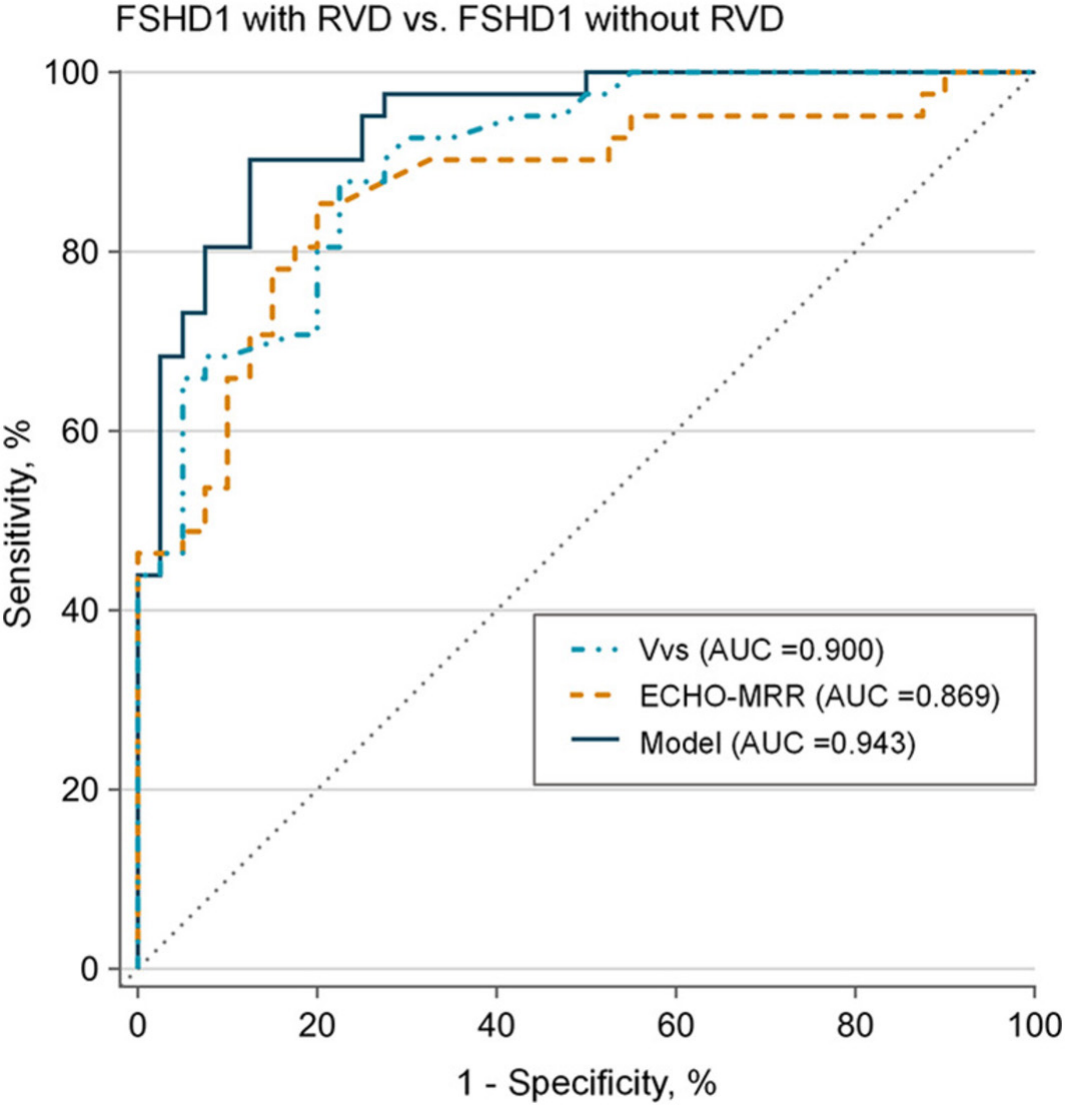
Receiver operating characteristic (ROC) curves for diaphragm ultrasound parameters and a multivariate model for identifying restrictive ventilatory defect (RVD) among FSHD1 patients. The multivariable logistic regression model (adjusted for age at examination, gender, D4Z4 repeat units, *V*_VS_ and ECHO‐MRR) was used to identify RVD in FSHD1 patients. AUC indicates area under the curve. The dashed diagonal line is a reference line representative of a test with no discriminatory ability (AUC = 0.500).

An ROC analysis of the right diaphragm ultrasound parameters indicated a relatively large AUC for predicting RVD in FSHD1 patients, ranging from 0.681 to 0.900 (Table S3). In particular, *V*_VS_ and ECHO‐MRR performed well in identifying RVD for FSHD1 patients, with AUC (95% CI) of 0.900 (0.831–0.961) and 0.869 (0.780–0.949) (both *p* < 0.0001) (Figure 2). The corresponding cut‐off values for *V*_VS_ and ECHO‐MRR of the right hemidiaphragm were 68.6 and 37.6 mm/s, respectively. Further, a multivariate model (including gender, age at examination, D4Z4 RUs, *V*_VS_, and ECHO‐MRR variables) for identifying RVD in the ROC curve analysis outperformed univariate models of *V*_VS_ and ECHO‐MRR, with AUC (95% CI) of 0.943 (0.896–0.989) (*p* < 0.0001) (Figure 2).

Moreover, we used *V*_VS_ and ECHO‐MRR cut‐off values of the right hemidiaphragm obtained from the ROC analysis above to assess the sensitivity of ultrasound parameters for identifying RVD in the FSHD1 validation cohort (*n* = 28) (Table S4). We observed a high true positive rate (80.0% and 80.0% by *V*_VS_ and ECHO‐MRR, respectively) and true negative rate (94.4% and 77.8% by *V*_VS_ and ECHO‐MRR, respectively) in identifying FSHD1 with RVD (Figure S2). Additionally, no differences in FVC% predicted and FEV1% predicted were detected between seated and supine PFTs for seven patients of the validation cohort (Figure S3).

### 3.4 Association Between Diaphragm Involvement Assessed by Ultrasound and Disease Severity in FSHD1 Patients

We then investigated the association between diaphragm involvement assessed by ultrasound parameters and disease severity for FSHD1 patients (Table 3). In general, we observed mild and negative correlations between age‐corrected clinical severity scale (ACSS) value and the following ultrasound parameters of the right diaphragm: *V*_VS_ (Spearman's *r* = −0.252, *p* = 0.023), ECHO‐MRR (Spearman's *r* = −0.268, *p* = 0.015), Th_FRC_ (Spearman's *r* = −0.359, *p* = 0.001) and *Th*_TLC_ (Spearman's *r* = −0.274, *p* = 0.013). Similar correlations were also found between ACSS value and PFT parameters (Table S5). Additionally, multivariate linear regression models showed that lower *V*_VS_ (*β* = −1.686, *p* = 0.033) and lower ECHO‐MRR (*β* = −1.761, *p* = 0.047) of the right diaphragm were significantly associated with higher ACSS value in FSHD1 patients (Table S6).

**TABLE 3:** Correlations of diaphragm ultrasound parameters with disease severity in FSHD1.

Variables		Right hemidiaphragm				Left hemidiaphragm			
		*V*_VS_	ECHO‐MRR	Th_FRC_	Th_TLC_	*V*_VS_	ECHO‐MRR	Th_FRC_	Th_TLC_
Upper Extremity Strength	*r*	0.133	0.049	−0.019	**0.240**	0.175	0.029	0.077	**0.255**
	*p* value	0.236	0.661	0.864	**0.031**	0.178	0.795	0.496	**0.022**
Lower extremity strength	*r*	**0.593**	**0.443**	**0.328**	**0.381**	**0.391**	**0.313**	**0.242**	**0.325**
	*p* value	**< 0.0001**	**< 0.0001**	**0.003**	**< 0.0001**	**< 0.0001**	**0.004**	**0.030**	**0.003**
FSHD clinical score (CS)	*r*	**−0.263**	−0.200	−0.161	**−0.275**	−0.180	−0.131	0.086	**−0.228**
	*p* value	**0.018**	0.073	0.154	**0.013**	0.109	0.243	0.443	**0.040**
Clinical severity scale (CSS)	*r*	**−0.288**	**−0.300**	−0.115	**−0.261**	**−0.302**	**−0.294**	−0.073	−0.219
	*p* value	**0.009**	**0.007**	0.306	**0.019**	**0.006**	**0.008**	0.514	0.050
Age‐corrected CSS (ACSS)	*r*	**−0.252**	**−0.268**	**−0.359**	**−0.274**	−0.183	−0.138	**−0.263**	−0.216
	*p* value	**0.023**	**0.015**	**0.001**	**0.013**	0.102	0.219	**0.018**	0.052

*Note:* The Spearman correlation coefficient was performed between variables. Values in bold indicate *p* values of < 0.05.

Given that early onset FSHD1 has been reported to associate with more severe phenotypes and late onset with milder phenotypes [22], we compared the diaphragm ultrasound parameters among FSHD1 patients stratified by different age at onset groups in the exploration cohort. We found that all the right hemidiaphragm ultrasound parameters were significantly lower in early onset FSHD1 patients than in typical onset patients (all *p* < 0.05) (Table S7). Compared to the control participants, the right hemidiaphragm ultrasound parameters of *V*_VS_ and ECHO‐MRR were significantly lower in late‐onset FSHD1 patients (Table S8). Additionally, early‐onset FSHD1 patients had a significantly higher frequency of RVD (14/19, 73.7%) than non‐early‐onset patients (27/62, 43.5%; *p* = 0.022; Figure S4A). Moderate‐severe RVD was more common in the early onset group, whereas only mild and moderate RVD were observed in the late onset group (Figure S4B,C).

**FIGURE 3:**
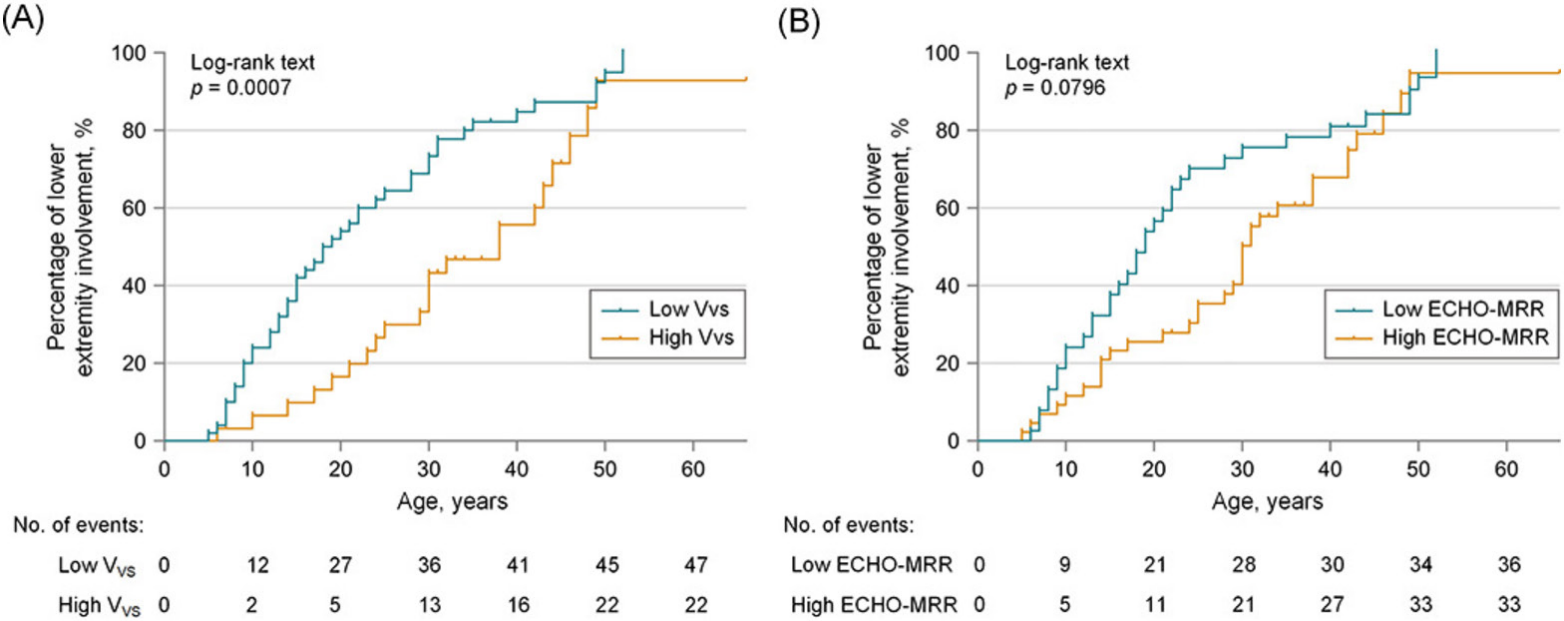
Kaplan–Meier curves illustrating outcome of lower extremity (LE) involvement in FSHD1 patients stratified by diaphragm ultrasound parameters. (A) Significant difference in outcome for LE involvement between FSHD1 patients with low *V*_VS_ and high *V*_VS_ was analysed by the log‐rank (Mantel–Cox) test (*p* = 0.0007). (B) No significant difference in outcome for LE involvement between FSHD1 patients with low ECHO‐MRR and high ECHO‐MRR (*p* = 0.0796).

Notably, we observed moderate and positive correlations between LE strength and two right diaphragm ultrasound parameters: *V*_VS_ (Spearman's *r* = 0.593, *p* < 0.0001) and ECHO‐MRR (Spearman's *r* = 0.443, *p* < 0.0001) (Table 3). Thus, we further explored LE involvement between FSHD1 patients with high and low diaphragm ultrasound parameters (*V*_VS_ and ECHO‐MRR). The Kaplan–Meier curve indicated that patients of the low *V*_VS_ subgroup were associated with an earlier onset age of LE involvement compared to those in the high *V*_VS_ subgroup (*p* = 0.0007) (Figure 3). After adjusting for gender, age at examination and number of D4Z4 RUs, a Cox regression showed that FSHD1 patients of the low *V*_VS_ subgroup and low ECHO‐MRR subgroup were at significantly higher risk of developing LE involvement, with aHRs of 2.353 (1.356–4.085, *p* = 0.002) and 2.039 (1.186–3.504, *p* = 0.010), respectively (Table S9).

## 4 Discussion

This study prospectively evaluated ultrasound technology for assessing diaphragm involvement in FSHD1 patients and examined the potential association of diaphragm involvement with respiratory involvement and disease severity. We found that diaphragm ultrasound parameters, including *V*_VS_ and ECHO‐MRR, can effectively identify FSHD1 patients with RVD. Moreover, the lower diaphragm ultrasound parameter values are associated with higher age‐corrected CCS values and with an earlier onset age of LE involvement among FSHD1 patients. These findings add to the understanding of the role of diaphragm involvement in FSHD1 and provide an imaging prognosis biomarker for clinicians.

Diaphragm ultrasound is understood as a noninvasive, direct measure of diaphragm function, and recent studies have reported that diaphragm ultrasound can be used to detect diaphragm involvement in neuromuscular diseases [28, 29]. Using a mouse model for Duchenne muscular dystrophy (DMD), a study found that diaphragm ultrasound supports in vivo evaluation of diaphragm function and reported that diaphragm excursion values were positively correlated with other measurements made for ex vivo force values while also being negatively correlated with the extent of diaphragm fibrosis [30]. An early study examining transdiaphragmatic pressure measurements reported that the dystrophic process in FSHD1 patients does not obviously involve the diaphragm muscles [31]. However, a more recent study using diaphragm ultrasound on 14 FSHD1 patients and 14 controls identified diaphragm weakness in FSHD1 patients [19]. In the present study, based on a larger sample of FSHD1 patients and controls, we found that all diaphragm ultrasound parameters were lower in FSHD1 patients compared to controls, confirming the diaphragm muscle weakness in FSHD1. Our data found no significant difference in the ultrasound comparison of left and right diaphragmatic impairment between FSHD1 patients and controls. This might be due to the fact that anatomical differences between the left and right diaphragms are common in the general population. Therefore, diaphragmatic asymmetry may not be considered a distinctive feature of FSHD1. Also, asymmetric diaphragm involvement has been reported in Pompe disease and other NMDs [32, 33].

The diaphragm is the principal muscle of respiration, and its contraction is essential for ventilation; any disease (such as COVID‐19 and DMD) that interferes with contractile muscle function can impair diaphragm function, leading to (for example) respiratory involvement, intolerance to exercise and even respiratory failure [17, 18, 34]. In FSHD1 respiratory assessment, PFT is currently the most widely used method for assessing respiratory function, and RVD is the most common pattern of respiratory involvement among FSHD1 patients [3, 4, 5]. We found that diaphragm parameters obtained via ultrasound showed a strong positive correlation with the PFT parameter of FVC% predicted, supporting previous findings [35]. Notably, there are potential limitations in assessing respiratory function in FSHD patients that should be considered: owing to the characteristic facial muscle weakness (~70%) in FSHD patients, issues like weakened bite force and air leakage could lead to inaccurate PFT results. We found all diaphragm ultrasound parameters in FSHD1 patients without RVD were significantly lower than those in control participants, suggesting the potential of diaphragm ultrasound for identifying early respiratory involvement in FSHD1 (potentially as well as other neuromuscular diseases in future studies).

Diaphragm ultrasound is understood as valuable for predicting RVD. For example, a study examined DMD patients with diaphragm ultrasound and reported that diaphragm excursion and diaphragm thickening rates (DTRs) are informatively predictive for RVD status [18]. In the present study, we found that the diaphragm parameter *V*_VS_ showed significant utility for identifying/predicting RVD (AUC of 0.900) and a multivariate model combining *V*_VS_ and ECHO‐MRR effectively identified RVD in FSHD1 patients, achieving a higher AUC of 0.943.

Previous studies have revealed an association between respiratory involvement and disease severity in FSHD1, showing a negative correlation between CSS score and PFT values of FVC [3, 4]; no informative correlation was detected between the CSS score and diaphragm excursion amplitude (*E*_TLC_) or DTR [19]. In the present study, we found that diaphragm ultrasound parameters including *V*_VS_ and ECHO‐MRR were negatively correlated with age‐corrected CSS. Furthermore, ultrasound parameters of the right hemidiaphragm demonstrated higher correlation coefficients with clinical severity than those of the left side, suggesting their potential utility in future clinical evaluations of disease severity in FSHD1. Previous studies have reported that *V*_VS_ and ECHO‐MRR were positively correlated with sniff nasal inspiratory pressure (SNIP) [36]. SNIP has been utilized to assess inspiratory muscle strength and weakness in patients with DMD [37]. Changes in diaphragm strength lead to changes in pressure [10, 35], and *V*_VS_ and ECHO‐MRR are useful as parameters for the muscle strength of the diaphragm. These findings argue that—rather than focusing solely on diaphragm muscle mass (such as thickening)—diaphragm muscle strength in FSHD1 should receive more attention in future clinical research.

Lower extremity involvement is understood as a prerequisite leading to independent ambulation loss and eventual progression to disability/wheelchair dependence in FSHD1 [38]. Previous studies have reported that severe respiratory involvement is frequently observed in wheelchair‐dependent FSHD1 patients [3, 4]. We observed moderate correlations between *V*_VS_ and ECHO‐MRR with LE muscle strength. Additionally, when stratifying FSHD1 patients into high and low *V*_VS_ subgroups to analyse LE involvement, we found that FSHD1 patients with low *V*_VS_ had a higher risk of developing LE involvement compared to FSHD1 patients with high *V*_VS_. These findings suggest that diaphragm ultrasound parameters could serve as potential biomarkers for disease severity and prognosis in FSHD1.

Early‐onset FSHD1 patients have been reported to exhibit more severe manifestations of muscle weakness, including greater facial muscle involvement and higher frequency of scoliosis [22, 39]. Our ultrasound data revealed that early‐onset FSHD1 patients also demonstrate more severe diaphragmatic impairment, manifested through significantly reduced right hemidiaphragm ultrasound parameters and increased frequency/severity of RVD compared to typical onset patients. These findings underscore the importance of early diaphragm ultrasound assessment in this population for respiratory function evaluation and treatment strategy guidance. In contrast, as late‐onset FSHD1 has been reported to exhibit milder phenotypes [22], we observed that only *V*_VS_ and ECHO‐MRR parameters of the right diaphragm showed significantly decreased values compared to controls. Given the limited sample size of late onset cases (*n* = 9) in our study, although exclusively mild and moderate RVD were observed in late onset patients, this still warrants ongoing respiratory surveillance for such patients. Future large‐scale studies with longitudinal respiratory function assessments will further elucidate the respiratory involvement progression patterns in FSHD1.

The current study has several limitations. First, owing to its prospective study design and the limited follow‐up period, we were unable to determine whether patients without RVD or those with lower *V*_VS_ values would eventually develop more severe clinical outcomes (e.g., loss of independent ambulation or wheelchair dependency). Second, we did not investigate whether asymptomatic FSHD1 carriers have underlying diaphragm involvement, making it unclear whether diaphragm involvement precedes or follows limb muscle weakness. This question could be further explored in future cohort studies within FSHD1 families. Third, previous research has indicated that PFTs in the supine position are more sensitive for diaphragm involvement than PFTs in the seated position [10]. We conducted a small study (*n* = 7) and found no significant differences in diaphragm involvement assessed from PFTs performed in seated versus supine positions; however, this is a limited sample size, and a larger‐scale assessment would be needed before reaching any conclusions about any potential systematic bias of PFT position. Finally, our study lacked severely wheelchair‐dependent patients, so we cannot determine the extent of diaphragm involvement in FSHD1 patients with severe symptoms.

## 5 Conclusions

In summary, our study suggests that values of *V*_VS_ and ECHO‐MRR obtained using noninvasive, accessible diaphragm ultrasound technology can serve as an informative imaging biomarker to estimate disease severity and disease progression. Further, our findings support that diaphragm ultrasound can be understood as a useful auxiliary tool to identify diaphragm involvement–induced RVD, especially for some patients with facial muscle dysfunction who cannot perform PFT, as with FSHD1, cerebral stroke, and other patients in intensive care units.

## Conflicts of Interest

The authors declare no conflicts of interest.

## Supporting information


**Data S1** Supporting Information.

**Table S1** Baseline characteristics of FSHD1 patients and control participants.
**Table S2** Correlations between bilateral diaphragm ultrasound parameters and PFT parameters.
**Table S3** Diaphragm ultrasound parameters of the right hemidiaphragm in the identification of RVD among FSHD1 patients.
**Table S4** Baseline characteristics and diaphragm ultrasound parameters of FSHD1 patients in the validation cohort.
**Table S5** Correlations between PFT parameters and disease severity in FSHD1.
**Table S6** Association between right hemidiaphragm diaphragm ultrasound parameters and disease severity in FSHD1.
**Table S7** Differences of diaphragm ultrasound parameters between early‐onset FSHD1 patients and typical onset FSHD1 patients in the exploration cohort.
**Table S8** Differences of diaphragm ultrasound parameters between late onset FSHD1 patients and control participants in the exploration cohort.
**Table S9** Cox regression analysis for diaphragm ultrasound parameters and lower extremity involvement of FSHD1 patients.
**Figure S1** Flowchart of the current study.
**Figure S2** Confusion matrix employed to evaluate the performance metrics of the diaphragm ultrasound parameters in identifying RVD in the FSHD1 validation cohort (*n* = 28).
**Figure S3** PFT conducted in both the seated and supine positions for the FSHD1 validation cohort (*n* = 7).
**Figure S4** Frequency of restrictive ventilatory defect (RVD) among FSHD1 patients stratified by different age at onset (AAO).

## Data Availability

The anonymized data that support the findings of this study are available from the corresponding author upon reasonable request from any qualified investigator.
