# Supplementary material for: Association of Diaphragm Involvement Assessed by Ultrasound With Disease Severity in Facioscapulohumeral Muscular Dystrophy
Source: J Cachexia Sarcopenia Muscle. 2025 Sep 12;16(5):e70057. doi: 10.1002/jcsm.70057 (PMC12426622; doi:10.1002/jcsm.70057)
Supplement: Supplementary file 2 — Table S1 Baseline characteristics of FSHD1 patients and control participants. Table S2 Correlations between bilateral diaphragm ultrasound parameters and PFT parameters. Table S3 Diaphragm ultrasound parameters of the right hemidiaphragm in the identification of RVD among FSHD1 patients. Table S4 Baseline characteristics and diaphragm ultrasound parameters of FSHD1 patients in the validation cohort. Table S5 Correlations between PFT parameters and disease severity in FSHD1. Table S6 Association between right hemidiaphragm diaphragm ultrasound parameters and disease severity in FSHD1. Table S7 Differences of diaphragm ultrasound parameters between early‐onset FSHD1 patients and typical onset FSHD1 patients in the exploration cohort. Table S8 Differences of diaphragm ultrasound parameters between late onset FSHD1 patients and control participants in the exploration cohort. Table S9 Cox regression analysis for diaphragm ultrasound parameters and lower extremity involvement of FSHD1 patients. Figure S1 Flowchart of the current study. Figure S2 Confusion matrix employed to evaluate the performance metrics of the diaphragm ultrasound parameters in identifying RVD in the FSHD1 validation cohort (n = 28). Figure S3 PFT conducted in both the seated and supine positions for the FSHD1 validation cohort (n = 7). Figure S4 Frequency of restrictive ventilatory defect (RVD) among FSHD1 patients stratified by different age at onset (AAO). [file JCSM-16-e70057-s001.docx]

**Supplementary Information for “Association of Diaphragm Involvement Assessed by Ultrasound with Disease Severity in Facioscapulohumeral Muscular Dystrophy”**

**Supplementary Table list:**

1. Table S1 Baseline Characteristics of FSHD1 patients and control participants
2. Table S2 Correlations between bilateral diaphragm ultrasound parameters and PFT parameters
3. Table S3 Diaphragm ultrasound parameters of the right hemidiaphragm in the identification of RVD among FSHD1 patients.
4. Table S4 Baseline characteristics and diaphragm ultrasound parameters of FSHD1 patients in the validation cohort.
5. Table S5 Correlations between PFT parameters and disease severity in FSHD1
6. Table S6 Association between right hemidiaphragm diaphragm ultrasound parameters and disease severity in FSHD1
7. Table S7 Differences of diaphragm ultrasound parameters between early-onset FSHD1 patients and typical onset FSHD1 patients in the exploration cohort
8. Table S8 Differences of diaphragm ultrasound parameters between late onset FSHD1 patients and control participants in the exploration cohort
9. Table S9 Cox regression analysis for diaphragm ultrasound parameters and lower extremity involvement of FSHD1 patients

**Supplementary Figure list:**

1. Figure S1 Flowchart of the current study.
2. Figure S2 Confusion Matrix employed to evaluate the performance metrics of the diaphragm ultrasound parameters in identifying RVD in FSHD1 validation cohort (*n* = 28).
3. Figure S3 PFT conducted in in both the seated and supine positions for the FSHD1 validation cohort (*n* = 7).
4. Figure S4 Frequency of restrictive ventilatory defect (RVD) among FSHD1 patients stratified by different age at onset (AAO).

**Supplementary Tables**

**Table S1 Baseline Characteristics of FSHD1 patients and control participants**

|  | **FSHD1 patients (*N* = 81)** | **Control participants (*N*=162)** | ***p* value^a^** |
| --- | --- | --- | --- |
| **Demographics** |  |  |  |
| Male, n (%) | 48 (59.3) | 96 (59.3) | 1.000 |
| Age at examination, y, median (IQR) | 33 (23, 43) | 31 (23, 45) | 0.899 |
| Height, cm, median (IQR) | 166 (159, 172) | 166 (160, 171) | 0.719 |
| Weight, kg, median (IQR) | 60.0 (51.0, 66.0) | 62.0 (57.0, 67.0) | **0.024** |
| BMI, kg·m^−2^, median (IQR) | 20.8 (18.9, 22.6) | 22.6 (21.8, 23.1) | **< 0.0001** |
| **Bilateral diaphragm ultrasound parameters, median (IQR)** |  |  |  |
| Right hemidiaphragm |  |  |  |
| V_VS_, mm/s | 63.3 (50.9, 73.8) | 89.0 (84.0, 93.3) | **< 0.0001** |
| ECHO-MRR, mm/s | 38.3 (24.1, 46.9) | 65.0 (57.0, 71.0) | **< 0.0001** |
| E_TLC_, mm, | 66.1 (56.1, 76.7) | 75.2 (70.0, 79.9) | **< 0.0001** |
| Th_FRC_, mm | 1.1 (0.9, 1.3) | 1.4 (1.3, 1.6) | **< 0.0001** |
| Th_TLC_, mm | 2.4 (2.0, 3.4) | 3.7 (3.3, 4.3) | **< 0.0001** |
| DTR | 2.4 (1.8, 2.8) | 2.6 (2.5, 2.8) | **< 0.0001** |
| Left hemidiaphragm |  |  |  |
| V_VS_, mm/s | 61.8 (43.7, 79.9) | 86.5 (81.0, 91.0) | **< 0.0001** |
| ECHO-MRR, mm/s | 22.1 (14.0, 40.0) | 63.0 (57.0, 69.0) | **< 0.0001** |
| E_TLC_, mm | 57.6 (43.3, 70.8) | 76.4 (70.7, 81.7) | **< 0.0001** |
| Th_FRC_, mm | 1.0 (0.9, 1.2) | 1.3 (1.2, 1.5) | **< 0.0001** |
| Th_TLC_, mm | 2.3 (1.9, 3.0) | 3.7 (3.3, 4.0) | **< 0.0001** |
| DTR | 2.3 (1.7, 2.9) | 2.7 (2.6, 2.8) | **< 0.0001** |
| **Pulmonary function tests (PFTs), median (IQR)** |  |  |  |
| FVC%predicted | 84.0 (70.5. 89.9) | 90.0 (87.0, 94.0) | **< 0.0001** |
| FEV1%predicted | 81.0 (71.5, 87.0) | 87.0 (83.0, 90.0) | **< 0.0001** |
| **Genetic characteristics** |  |  |  |
| Number of contracted D4Z4 repeat units, median (IQR) | 5 (4, 6) |  |  |
| **Clinical assessments** |  |  |  |
| Age at onset, y, median (IQR) | 15 (10, 20) |  |  |
| Medical Research Council (MRC) score, median (IQR) |  |  |  |
| Upper Extremity strength | 56.0 (47.0, 61.0) |  |  |
| Lower Extremity strength | 58.5 (47.0, 66.0) |  |  |
| Clinical severity/progression assessments, median (IQR) |  |  |  |
| FSHD clinical score (CS, 0–15) | 7.0 (5.0, 9.0) |  |  |
| Clinical severity scale (CSS, 0–5) | 3.0 (2.5, 3.5) |  |  |
| Age-corrected CSS (ACSS, 0–10,000) | 176.5 (125.1, 283.5) |  |  |

^a^Mann-Whitney U test or χ^2^ test was used.

Values in bold indicate *p* value < 0.05.

**Table S2 Correlations between bilateral diaphragm ultrasound parameters and PFT parameters**

| **Parameters** | **FVC%predicted** | | **FEV1%predicted** | |
| --- | --- | --- | --- | --- |
|  | **r** | ***p* value** | **r** | ***p* value** |
| Right hemidiaphragm | | | | |
| V_VS_, mm/s, mean (SD) | **0.639** | **< 0.0001** | **0.622** | **< 0.0001** |
| ECHO-MRR, mm/s, mean (SD) | **0.521** | **< 0.0001** | **0.500** | **< 0.0001** |
| E_TLC_, mm, mean (SD) | **0.335** | **0.002** | **0.294** | **0.008** |
| Th_FRC_, mm, mean (SD) | 0.097 | 0.387 | 0.115 | 0.167 |
| Th_TLC_, mm, mean (SD) | **0.502** | **< 0.0001** | **0.548** | **< 0.0001** |
| DTR | **0.413** | **< 0.0001** | **0.444** | **< 0.0001** |
| Left hemidiaphragm | | | | |
| V_VS_, mm/s, mean (SD) | **0.485** | **< 0.0001** | **0.404** | **< 0.0001** |
| ECHO-MRR, mm/s, mean (SD) | **0.420** | **< 0.0001** | **0.357** | **< 0.0001** |
| E_TLC_, mm, mean (SD) | **0.388** | **< 0.0001** | **0.283** | **0.010** |
| Th_FRC_, mm, mean (SD) | 0.141 | 0.211 | 0.102 | 0.363 |
| Th_TLC_, mm, mean (SD) | **0.404** | **< 0.0001** | **0.391** | **< 0.0001** |
| DTR | **0.330** | **0.003** | **0.368** | **0.001** |

**Table S3 Diaphragm ultrasound parameters of the right hemidiaphragm in the identification of RVD among FSHD1 patients**

|  | **AUC (95% CI)** | ***p* value** | **Sensitivity** | **Specificity** |
| --- | --- | --- | --- | --- |
| V_VS_, mm/s^a^ | 0.900 (0.831-0.961) | **< 0.0001** | 0.700 | 0.927 |
| ECHO-MRR, mm/s^a^ | 0.868 (0.780-0.949) | **< 0.0001** | 0.850 | 0.780 |
| E_TLC_, mm | 0.681 (0.565-0.797) | **0.005** | 0.463 | 0.900 |
| Th_TLC_, mm | 0.785 (0.681-0.888) | **< 0.0001** | 0.707 | 0.825 |
| DTR | 0.769 (0.666-0.872) | **< 0.0001** | 0.634 | 0.825 |
| Multivariable model^a^ | 0.943 (0.896-0.989) | **< 0.0001** |  |  |

^a^The different multivariable logistic regression models (adjusted by age at examination, gender, D4Z4 repeat units, V_VS_ and ECHO-MRR) identify RVD among FSHD1 patients with p value and OR for each variable. Sensitivity and specificity values were calculated using the Youden Index.

Abbreviations: RVD = restrictive ventilatory defect; ID = identification; AUC = area under the receiver operating characteristic curve

**Table S4 Baseline characteristics and diaphragm ultrasound parameters of FSHD1 patients in the validation cohort**

|  | **FSHD1 validation cohort (*N* = 28)** |
| --- | --- |
| **Demographics** |  |
| Male, n (%) | 16 (57.1) |
| Age at examination, y, median (IQR) | 33.5 (21.5, 38.5) |
| Height, cm, median (IQR) | 162.0 (158, 173.5) |
| Weight, kg, median (IQR) | 54.3 (45.5, 67.0) |
| BMI, kg·m^−2^, median (IQR) | 20.0 (16.7, 23.9) |
| **Bilateral diaphragm ultrasound parameters, median (IQR)** | |
| Right hemidiaphragm |  |
| V_VS_, mm/s | 77.2 (66.5, 98.7) |
| ECHO-MRR, mm/s | 40.0 (19.8, 65.5) |
| E_TLC_, mm, | 60.4 (56.2, 71.0) |
| Th_FRC_, mm | 1.1 (1.0, 1.4) |
| Th_TLC_, mm | 3.3 (2.7, 3.8) |
| DTR | 2.7 (2.4, 3.4) |
| Left hemidiaphragm |  |
| V_VS_, mm/s | 70.3 (60.0, 91.6) |
| ECHO-MRR, mm/s | 24.7 (19.7, 46.6) |
| E_TLC_, mm | 54.9 (39.4, 75.4) |
| Th_FRC_, mm | 1.0 (0.8, 1.1) |
| Th_TLC_, mm | 2.7 (1.9, 3.5) |
| DTR | 2.5 (2.3, 3.1) |
| **Pulmonary function tests (PFTs), median (IQR)** | |
| FVC%predicted | 88.3 (70.6, 94.2) |
| FEV1%predicted | 86.7 (73.8, 93.8) |
| **Genetic characteristics** |  |
| Number of contracted D4Z4 repeat units, median (IQR) | 5 (4, 6) |
| **Clinical assessments** |  |
| Age at onset, y, median (IQR) | 13 (10.5, 19) |
| Early-onset, n (%) | 6 (21.4) |
| Medical Research Council (MRC) score, median (IQR) |  |
| Upper Extremity strength | 62.8 (49.8, 66.5) |
| Lower Extremity strength | 52.0 (46.0, 63.3) |
| Clinical severity/progression assessments, median (IQR) |  |
| FSHD clinical score (CS, 0–15) | 8.5 (5.5, 10.0) |
| Clinical severity scale (CSS, 0–5) | 3.0 (3.0, 4.0) |
| Age-corrected CSS (ACSS, 0–10,000) | 179.1 (130.3, 227.8) |

**Table S5 Correlations between PFT parameters and disease severity in FSHD1**

| **Parameters** | **FVC%predicted** | | **FEV1%predicted** | |
| --- | --- | --- | --- | --- |
|  | **r** | ***p* value** | **r** | ***p* value** |
| Upper Extremity Strength | **0.360** | **0.001** | **0.240** | **0.031** |
| Lower Extremity Strength | **0.462** | **< 0.0001** | **0.495** | **< 0.0001** |
| FSHD clinical score (CS) | **-0.374** | **0.001** | **-0.369** | **0.001** |
| Clinical severity scale (CSS) | **-0.468** | **< 0.0001** | **-0.432** | **< 0.0001** |
| Age-corrected CSS (ACSS) | **-0.375** | **0.001** | **-0.260** | **0.019** |

The Spearman correlation coefficient was performed between variables.

Values in bold indicate *p* value < 0.05.

**Table S6 Association between right hemidiaphragm diaphragm ultrasound parameters and disease severity in FSHD1**

|  | **Ultrasound parameters** | ***β*** | **95% CI** | ***p* value^a^** |
| --- | --- | --- | --- | --- |
| Lower Extremity Strength | V_VS_ | **0.341** | **0.204 - 0.479** | **< 0.0001** |
|  | ECHO-MRR | **0.349** | **0.191 - 0.507** | **< 0.0001** |
|  | Th_FRC_ | 10.183 | -1.564 - 21.930 | 0.088 |
|  | Th_TLC_ | **4.996** | **1.883 -** **8.109** | **0.002** |
| FSHD clinical score (CS) | V_VS_ | -0.032 | -0.064 - 0.001 | 0.055 |
|  | ECHO-MRR | -0.032 | -0.068 - 0.005 | 0.086 |
|  | Th_FRC_ | -1.798 | -4.277 - 0.680 | 0.153 |
|  | Th_TLC_ | **-0.741** | **-1.146 - -0.066** | **0.032** |
| Clinical severity scale (CSS) | V_VS_ | -0.010 | -0.021 - 0.002 | 0.109 |
|  | ECHO-MRR | -0.012 | -0.025 - 0.001 | 0.081 |
|  | Th_FRC_ | -0.155 | -1.014 - 0.785 | 0.800 |
|  | Th_TLC_ | -0.189 | -0.434 - 0.056 | 0.129 |
| Age-corrected CSS (ACSS) | V_VS_ | **-1.686** | **-3.320 -** **-0.141** | **0.033** |
|  | ECHO-MRR | **-1.761** | **-3.497 -** **-0.026** | **0.047** |
|  | Th_FRC_ | -35.541 | -155.736 - 84.653 | 0.558 |
|  | Th_TLC_ | -20.528 | -53.55 - 12.494 | 0.219 |

^a^The multivariate linear models (adjusted for gender, age at examination, number of contracted D4Z4 repeat units) were performed to assess the association between force category parameters obtained by sniff ultrasound and disease severity in FSHD1.

Values in bold indicate *p* value < 0.05.

Abbreviation: FSHD1 = facioscapulohumeral muscular dystrophy type 1

**Table S7 Differences of diaphragm ultrasound parameters between early onset FSHD1 patients and typical onset FSHD1 patients in the exploration cohort**

|  | **Early onset  FSHD1  (*N* = 19)** | **Typical onset FSHD1 (*N* = 53)** | ***p* value^a^** |
| --- | --- | --- | --- |
| **Demographics** |  |  |  |
| Male, n (%) | 22 (55.0) | 33 (62.3) | 0.463 |
| Age at examination, y, median (IQR) | 14 (10, 28.5) | 34 (29, 43) | **0.001** |
| Height, cm, median (IQR) | 157.0 (147.5, 165.0) | 168.0 (162.0, 173.0) | **0.001** |
| Weight, kg, median (IQR) | 43.0 (40.5, 58.0) | 62.0 (55.0, 68.0) | **0.001** |
| BMI, kg·m^−2^, median (IQR) | 18.4 (17.4, 20.8) | 21.7 (20.1, 23.0) | **0.006** |
| **Bilateral diaphragm ultrasound parameters, median (IQR)** | | | |
| Right hemidiaphragm |  |  |  |
| V_VS_, mm/s | 52.3 (49.5, 61.4) | 68.4 (58.4, 75.1) | **0.001** |
| ECHO-MRR, mm/s | 32.2 (21.8, 39.9) | 40.4 (24.7, 47.5) | **0.022** |
| E_TLC_, mm, | 59.3 (43.8, 68.7) | 66.9 (58.9, 77.3) | **0.048** |
| Th_FRC_, mm | 1.0 (0.9, 1.1) | 1.2 (1.0, 1.3) | **0.049** |
| Th_TLC_, mm | 2.2 (1.9, 2.5) | 2.5 (2.1, 3.3) | **0.022** |
| DTR | 2.1 (1.8, 2.6) | 2.5 (1.8, 2.8) | 0.258 |
| Left hemidiaphragm |  |  |  |
| V_VS_, mm/s | 49.6 (34.8, 69.3) | 68.0 (50.5, 81.0) | 0.051 |
| ECHO-MRR, mm/s | 20.0 (10.7, 34.7) | 32.0 (19.1, 42.4) | **0.043** |
| E_TLC_, mm | 49.3 (40.2, 64.9) | 60.4 (50.0, 74.0) | **0.032** |
| Th_FRC_, mm | 1.0 (0.9, 1.1) | 1.0 (0.9, 1.2) | 0.638 |
| Th_TLC_, mm | 2.0 (1.8, 2.7) | 2.4 (2.0, 3.3) | 0.050 |
| DTR | 2.0 (1.6, 2.5) | 2.4 (2.0, 3.0) | 0.102 |
| **Pulmonary function tests (PFTs), median (IQR)** | | | |
| FVC%predicted | 71.4 (68.4, 86.5) | 85.5 (72.1, 91.5) | **0.015** |
| FEV1%predicted | 72.5 (67.5, 84.4) | 83.1 (75.0, 87.0) | **0.040** |
| **Genetic characteristics** |  |  |  |
| Number of contracted D4Z4 repeat units, median (IQR) | 4.0 (3.0, 4.5) | 5.0 (4.0, 6.0) | **< 0.0001** |
| **Clinical assessments** |  |  |  |
| Age at onset, y, median (IQR) | 4.0 (5.0, 6.5) | 15.0 (13.0, 20.0) | **< 0.0001** |
| Medical Research Council (MRC) score, median (IQR) |  |  |  |
| Upper Extremity strength | 54.5 (45.5, 59.3) | 55.5 (46.0, 62.0) | 0.482 |
| Lower Extremity strength | 47.0 (36.5, 62.3) | 60.5 (50.0, 66.0) | **0.009** |
| Clinical severity/progression assessments, median (IQR) |  |  |  |
| FSHD clinical score (CS, 0–15) | 7.0 (4.5, 9.0) | 8.0 (6.0, 9.0) | 0.620 |
| Clinical severity scale (CSS, 0–5) | 3.5 (3.0, 4.0) | 3.0 (2.5, 3.5) | **0.022** |
| Age-corrected CSS (ACSS, 0–10,000) | 421.1 (283.5, 666.7) | 129.0 (116.3, 227.3) | **< 0.0001** |

^a^Mann-Whitney *U* test or χ^2^ test was used.

Values in bold indicate *p* value < 0.05.

**Table S8 Differences of diaphragm ultrasound parameters between late onset FSHD1 patients and control participants in the exploration cohort**

|  | **Late onset  FSHD1 (*N* = 9)** | **Control participants (*N* = 162)** | ***p* value^a^** |
| --- | --- | --- | --- |
| **Demographics** |  |  |  |
| Male, n (%) | 5 (55.5) | 96 (59.3) | 0.826 |
| Age at examination, y, median (IQR) | 53 (52, 54) | 31 (23, 45) | **0.003** |
| Height, cm, median (IQR) | 166.0 (161.0, 169.0) | 166 (160, 171) | 0.964 |
| Weight, kg, median (IQR) | 60.0 (54.0, 63.0) | 62.0 (57.0, 67.0) | 0.352 |
| BMI, kg·m^−2^, median (IQR) | 20.8 (19.6, 22.9) | 22.6 (21.8, 23.1) | 0.197 |
| **Bilateral diaphragm ultrasound parameters, median (IQR)** | | | |
| Right hemidiaphragm |  |  |  |
| V_VS_, mm/s | 60.0 (48.5, 79.0) | 89.0 (84.0, 93.3) | **0.006** |
| ECHO-MRR, mm/s | 37.3 (30.0, 40.0) | 65.0 (57.0, 71.0) | **< 0.0001** |
| E_TLC_, mm, | 77.5 (70.3, 93.9) | 75.2 (70.0, 79.9) | 0.356 |
| Th_FRC_, mm | 1.2 (1.0, 1.3) | 1.4 (1.3, 1.6) | **0.025** |
| Th_TLC_, mm | 2.9 (1.9, 3.5) | 3.7 (3.3, 4.3) | **0.002** |
| DTR | 2.2 (1.4, 2.9) | 2.6 (2.5, 2.8) | 0.187 |
| Left hemidiaphragm |  |  |  |
| V_VS_, mm/s | 46.0 (40.0, 65.1) | 86.5 (81.0, 91.0) | **< 0.0001** |
| ECHO-MRR, mm/s | 18.9 (10.0, 20.0) | 63.0 (57.0, 69.0) | **< 0.0001** |
| E_TLC_, mm | 49.7 (45.4, 68.3) | 76.4 (70.7, 81.7) | **< 0.0001** |
| Th_FRC_, mm | 1.1 (1.0, 1.2) | 1.3 (1.2, 1.5) | **0.002** |
| Th_TLC_, mm | 2.8 (1.5, 3.0) | 3.7 (3.3, 4.0) | **< 0.0001** |
| DTR | 2.0 (1.3, 2.5) | 2.7 (2.6, 2.8) | **0.008** |
| **Pulmonary function tests (PFTs), median (IQR)** | | | |
| FVC%predicted | 84.2 (72.2, 86.4) | 90.0 (87.0, 94.0) | **0.013** |
| FEV1%predicted | 81.0 (70.7, 82.4) | 87.0 (83.0, 90.0) | **0.018** |
| **Genetic characteristics** |  |  |  |
| Number of contracted D4Z4 repeat units, median (IQR) | 7.0 (7.0, 8.0) |  |  |
| **Clinical assessments** |  |  |  |
| Age at onset, y, median (IQR) | 47.0 (34.0, 47.0) |  |  |
| Medical Research Council (MRC) score, median (IQR) |  |  |  |
| Upper Extremity strength | 59.5 (53.5, 60.5) |  |  |
| Lower Extremity strength | 59.5 (56.5, 68.0) |  |  |
| Clinical severity/progression assessments, median (IQR) |  |  |  |
| FSHD clinical score (CS, 0–15) | 6.0 (4.0, 6.0) |  |  |
| Clinical severity scale (CSS, 0–5) | 3.0 (2.5, 3.0) |  |  |
| Age-corrected CSS (ACSS, 0–10,000) | 115.4 (111.1, 121.2) |  |  |

^a^Mann-Whitney *U* test or χ^2^ test was used.

Values in bold indicate *p* value < 0.05.

**Table S9 Cox regression analysis for diaphragm ultrasound parameters and lower extremity involvement of FSHD1 patients**

| **Variable** | **Multivariate Cox model 1**a | | **Multivariate Cox model 2**b | |
| --- | --- | --- | --- | --- |
|  | **HR (95% CI)** | ***p* value** | **aHR (95% CI)** | ***p* value** |
| **V_VS_, mm/s** | | | | |
| Low V_VS_ | 2.353 (1.356-4.085) | 0.002 |  |  |
| High V_VS_ | reference |  |  |  |
| **ECHO-MRR, mm/s** | | | | |
| Low ECHO-MRR |  |  | 2.039 (1.186-3.504) | 0.010 |
| High ECHO-MRR |  |  | reference |  |
| **Sex** | | | | |
| Female | 0.972 (0.589-1.605) | 0.913 | 1.170 (0.692-1.979) | 0.558 |
| Male | reference |  | reference |  |
| **Age at examination** | 0.888 (0.858-0.920) | **< 0.0001** | 0.886 (0.856-0.917) | **< 0.0001** |
| **D4Z4 repeat units** | 0.837 (0.711-0.985) | **0.033** | 0.873 (0.737-1.033) | 0.114 |

^a^Multivariate Cox model 1 with variables of sex, age at examination, D4Z4 RUs, and diaphragm ultrasound parameter of V_VS_.

^b^Multivariate Cox model 2 with variables of sex, age at examination, D4Z4 RUs, and diaphragm ultrasound parameter of ECHO-MRR.

Values in bold indicate *p* value < 0.05.

Abbreviation: FSHD1 = facioscapulohumeral muscular dystrophy type 1; HR = hazard ratios; aHR = adjusted hazard ratios

**Supplementary Figure**


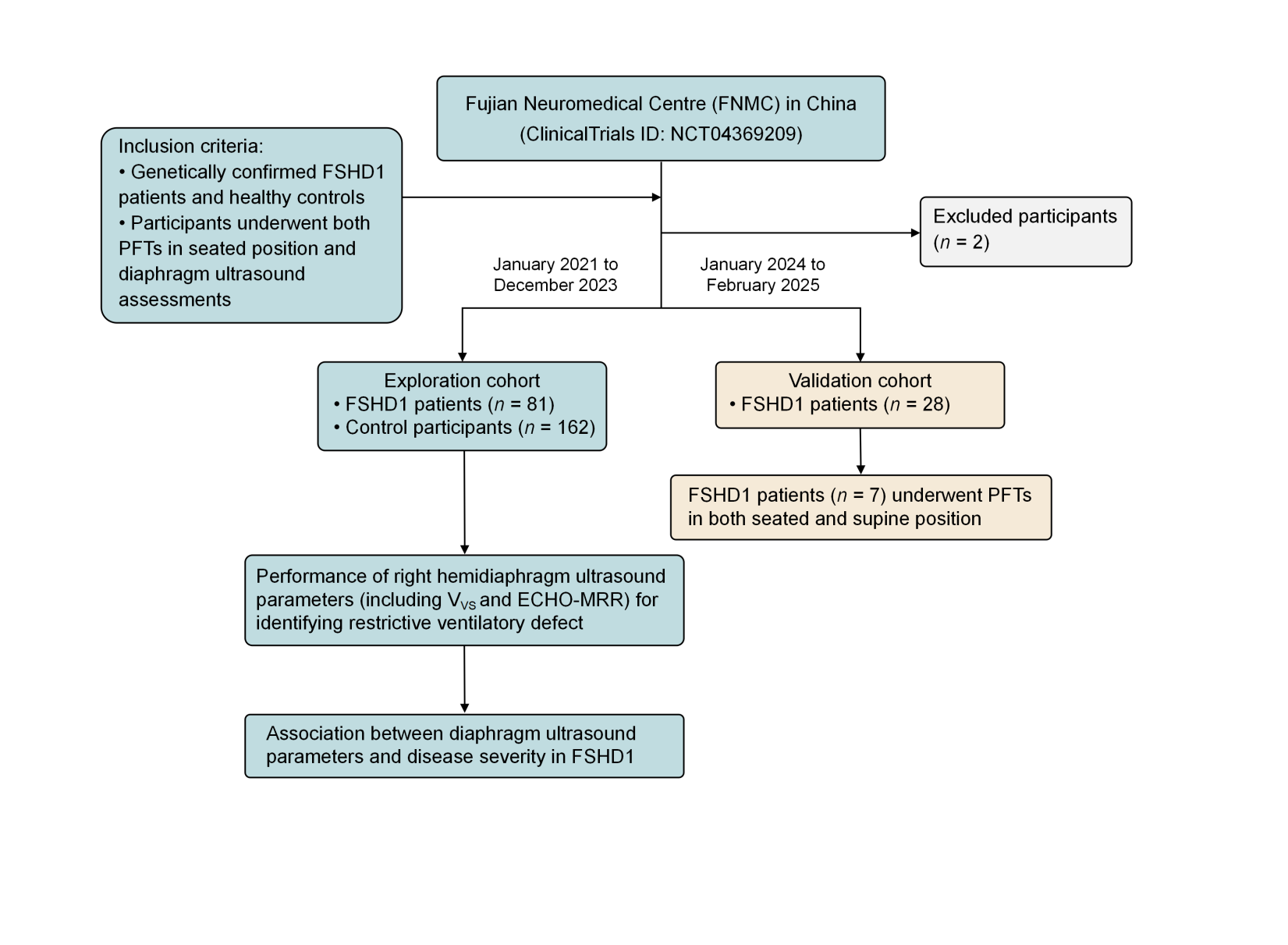


**Figure S1. Flowchart of the current study.** FSHD1, facioscapulohumeral muscular dystrophy type 1; PFTs, pulmonary function tests.


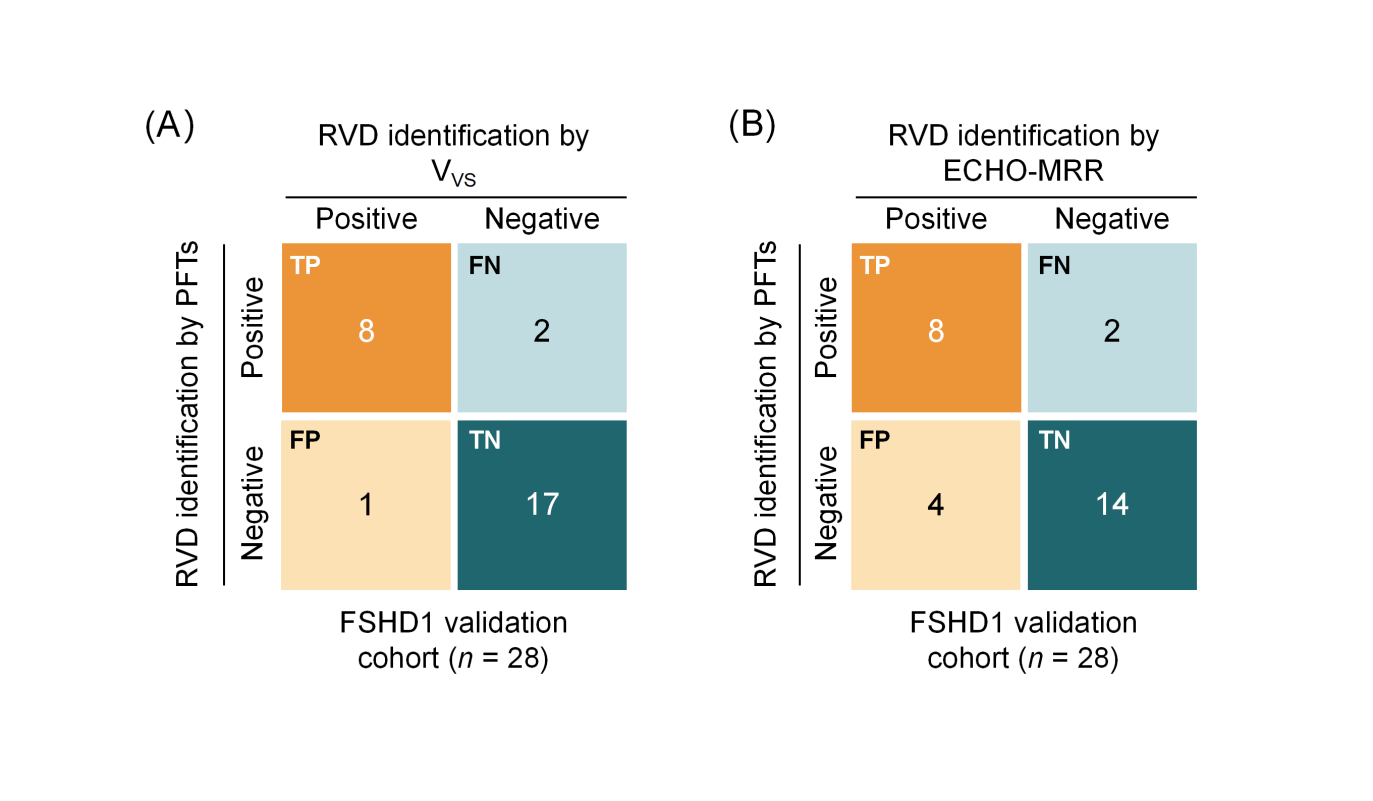


**Figure S2 Confusion Matrix employed to evaluate the performance metrics of the diaphragm ultrasound parameters in identifying RVD in FSHD1 validation cohort (*n* = 28).** The Confusion Matrix shows the number of True Positives (TP), False Positives (FP), True Negatives (TN), and False Negatives (FN) according to RVD identification based on (A) V_VS_ and (B)ECHO-MRR.


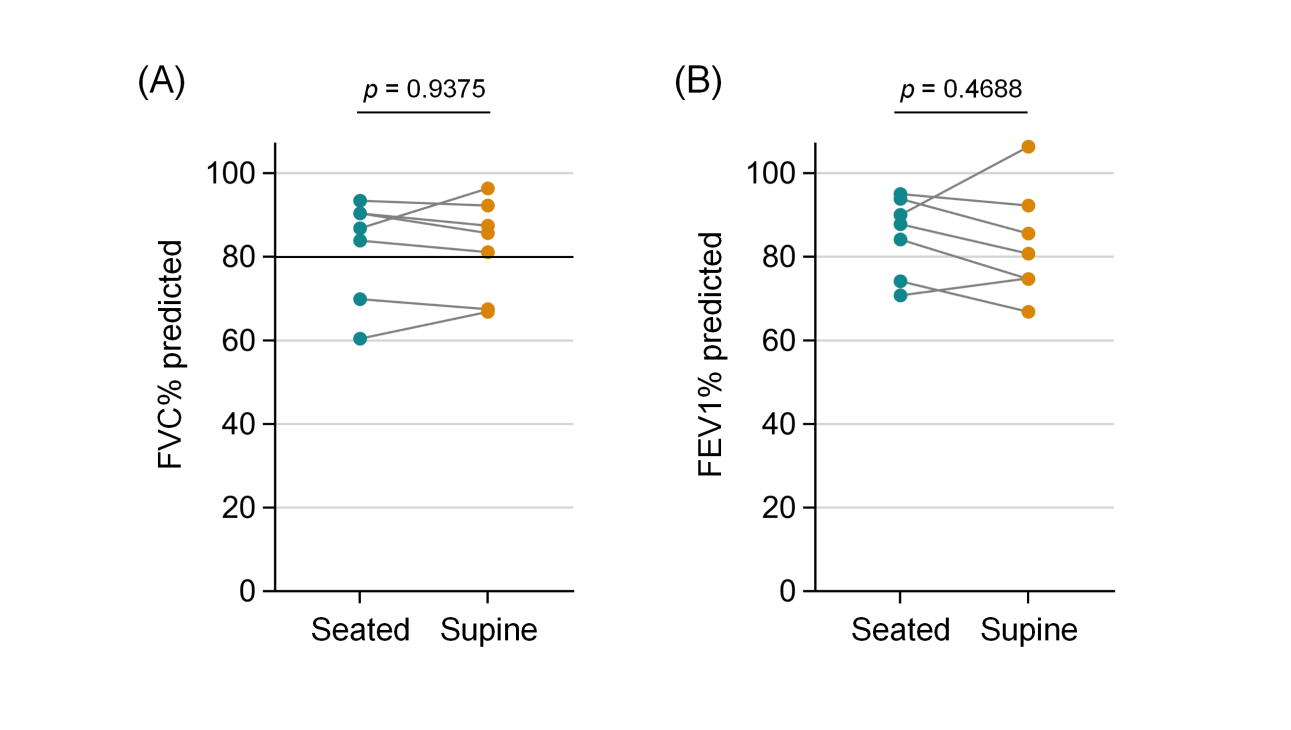


**Figure S3. PFT conducted in in both the seated and supine positions for the FSHD1 validation cohort (n = 7).** (A, B) No differences in (A) FVC% predicted and (B) FEV1% predicted between seated and supine PFTs were analyzed using a paired t-test.


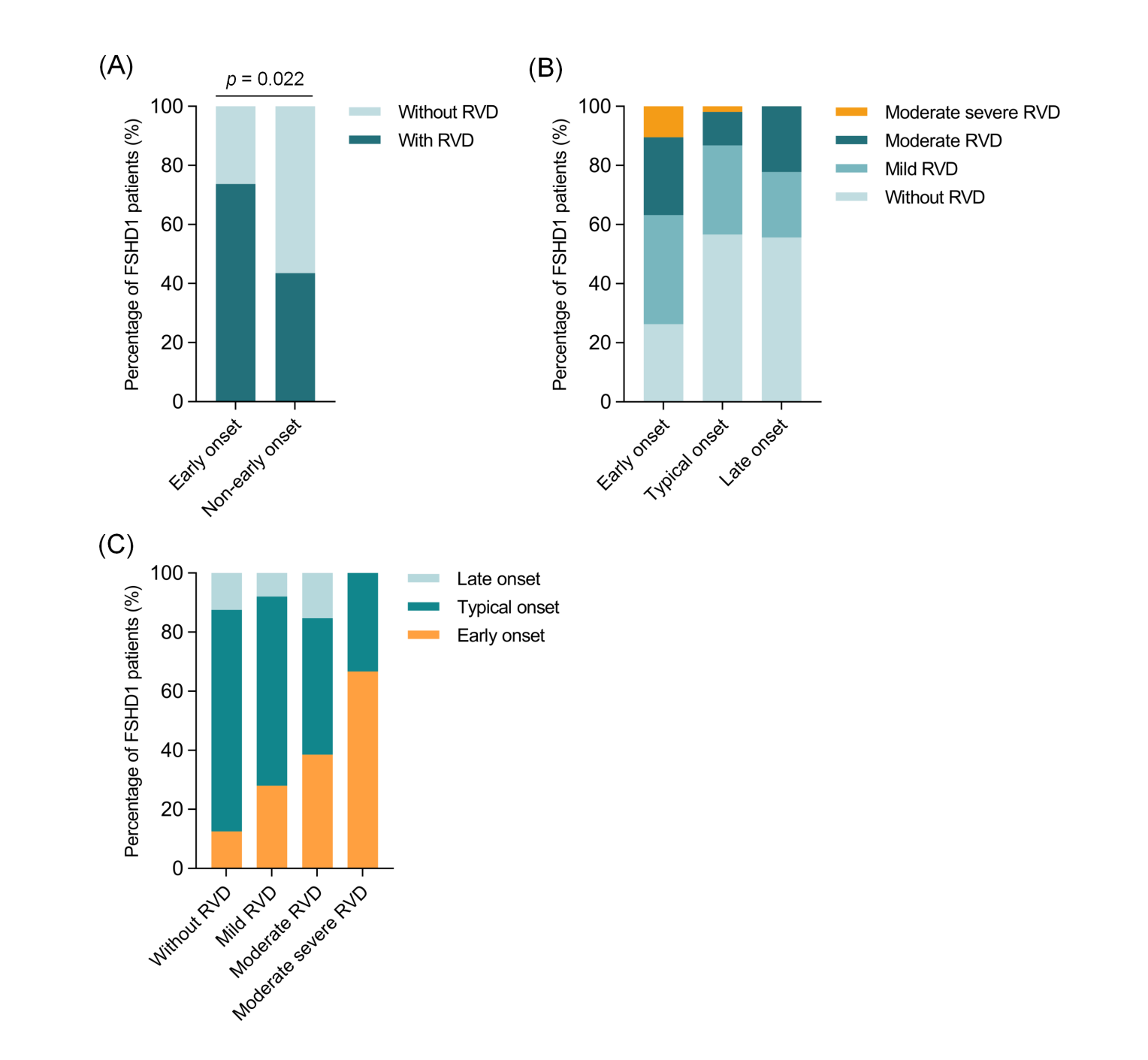


**Figure S4 Percentage of restrictive ventilatory defect (RVD) among FSHD1 patients stratified by different age at onset (AAO).** (A) Comparison of RVD percentage between early onset (AAO < 10 years) and non–early onset (AAO ≥ 10 years) FSHD1 patients. (B) Distribution of RVD grades among FSHD1 patients stratified into three AAO groups: early-onset (< 10 years, *n* = 19), typical onset (10–29 years, *n* = 53), and late onset (≥ 30 years, *n* = 9). (C) Distribution of AAO categories among FSHD1 patients stratified by RVD grades.
